# DYADIC PREDICTORS OF AGING SATISFACTION: RELATIONSHIP QUALITY MODULATES THE IMPACT OF HEALTH RESTRICTIONS

**DOI:** 10.1093/geroni/igz038.1716

**Published:** 2019-11-08

**Authors:** Daniela Jopp, Charikleia Lampraki, Claudia Meystre, Kyungmin Kim, Yijung K Kim, Kathrin Boerner

**Affiliations:** 1 University of Lausanne, Lausanne, Switzerland, Switzerland; 2 University of Lausanne, Institute of Psychology, National Pole of Research LIVES, Lausanne, Switzerland; 3 University of Lausanne, Institute of Psychology, Lausanne, Vaud, Switzerland; 4 University of Massachusetts Boston, Boston, Massachusetts, United States

## Abstract

How individuals develop perceptions of their own aging process is receiving increasing attention. While own age-related experiences are important, the aging of close others, such as parents, has been also found to play a role. Of particular interest may be parental health, yet relationship quality aspects may influence the extent to which health restrictions affect the children’s aging perceptions. Dyadic data from the Swiss “Aging Together” study were analyzed (dyad N = 98, Parent Mage = 83.85; Child Mage = 56.43). For parents, actor-partner interdependence models indicated a negative relationship between health restrictions and aging satisfaction; the more health restrictions they experienced, the less happy they were with their aging process. Parental health restrictions also played a role for children’s aging satisfaction, but the effect was dependent on how the relationship with the parent was perceived by the child; parents’ health restrictions were negatively associated with aging satisfaction of children who reported better relationship quality (i.e., less conflictual), but not with aging satisfaction of children who reported worse relationship quality (i.e., more conflictual). In addition, when the parent was more restricted, children who perceived the parent as more supportive showed lower levels of aging satisfaction, compared to children who perceived the parent as less supportive. Lastly, children’s own health restrictions had no negative impact on their aging satisfaction when they perceived the parent as more supportive. In sum, health restrictions may influence both dyadic partners’ aging satisfaction, but relationship quality modulates their impact.

